# microRNA-21 regulates astrocytic reaction post-acute phase of spinal cord injury through modulating TGF-β signaling

**DOI:** 10.18632/aging.101484

**Published:** 2018-06-23

**Authors:** Ronghan Liu, Wenzhao Wang, Shuya Wang, Wei Xie, Hongfei Li, Bin Ning

**Affiliations:** 1 Jinan Central Hospital Affiliated to Shandong University, Jinan 250013, Shandong, China; 2 Affiliated Hospital of Taishan Medical University, Taian 271000, Shandong, China

**Keywords:** spinal cord injury, microRNA-21, astrocytic scar, fibrotic scar, astrogliosis, TGF-β, glial fibrillary acidic protein, apoptosis

## Abstract

Astrogliosis following spinal cord injury (SCI) was considered as a negative factor for neural regeneration. We found that miR-21 was significantly upregulated after SCI. So, we aim to determine whether miR-21 acts in a positive manner post SCI. *In vitro*, we measured the proliferation, apoptosis and cytokine secretion of primary cultured astrocytes after modulating the expression of miR-21 by western blot, RT-PCR and immunofluorescence. *In vivo*, we performed a modified Allen’s weight drop model. Manipulation of the miR-21 expression level was achieved by interfering with antagomir and agomir. Clinic score was evaluated and recorded every day. Then, western blot, immunohistochemistry, TUNEL assay and ELISA were performed to detect pathological and functional alterations. Our results demonstrate that miR-21 can modulate the secretion, proliferation and apoptosis of astrocytes to promote recovery after SCI both *in vivo* and *in vitro*. These effects are likely mediated through transforming growth factor beta mediated targeting of the PI3K/Akt/mTOR pathway. These data suggest that miR-21 can regulate astrocytic function, then promote the functional recovery after SCI. We therefore highlight the positive effects of miR-21 after SCI.

## Introduction

Spinal cord injury (SCI) is the most severe complication of spine trauma because it causes serious physical and psychological harm. Many studies have suggested that astrogliosis following SCI resulted in local tissue hypertrophic response, limited inflammation, and subsequential formation of glial scars that may act as a physical barrier for axon regeneration [1,2]. However, more recent investigations indicated that astrogliosis might act positively after SCI [3]. Glial scar not only prevented inflammation, but could also provide favorable conditions for axon regeneration [3,4][REMOVED HYPERLINK FIELD]. In the chronic phase after SCI, astrocytic scarring was suggested to act as a barrier because of its interaction with fibroblasts [5], although recent studies found that removal of astrocytic scars from the lesion area did not improve axon regeneration [3], which probably due to the loss of the positive effect of astrocytic scars. Moreover, astrocytes play a critical role in the process of astrocytic scar formation. After injury, astrocytes become activated, characterized by increased expression of glial fibrillary acidic protein (GFAP). They become hypertrophic and hyperplasia occurs around the lesion site. Subsequently, they acquire stronger secretory functions, especially for chondroitin sulfate proteoglycans (CSPGs), which is a potent inhibitor of axonal regeneration [2,6-8]. However, beyond astrocytes, fibroblasts, pericytes and other inflammatory cells [9] could also produce CSPGs [10]. Additionally, secretion of neurotrophins such as brain-derived neurotrophic factor (BDNF) and nerve growth factor (NGF) has been identified in astrocytes [11]. In this regard, we hypothesized that astrocytes might play a dual role in axon regeneration during both acute and chronic stages of SCI.

In our current study, we first measured total mRNA and microRNA (miR) expression in spinal cord samples from the 3 days, when the glial scar formed, after SCI group, and compared them with a control group. After a microarray screen of miRs and mRNA, and statistical analysis, we found that many miRs and mRNA expression were changed. Among these, we found that miR-21 showed a significant up-regulation. Transforming growth factor-beta1 (TGF-β1), its receptor and its related proteins were upregulated after SCI, as previously demonstrated [12]. We also noticed the expression changes regarding the TGF-β1 related protein family and TGF-β1 could be produced by many types of cells, and is believed to promote cell proliferation and migration [13]. For instance, TGF-β1 activates glial cells and phagocytes to form connective tissue and extracellular matrix, which may play a key role during tissue repair post SCI [14,15]. We thus speculate that TGF-β1 might play critical roles in astrocyte hypertrophy and proliferation, and therefore, are important for both astrogliosis and astrocytic scar formation [16].

MicroRNAs (miRs) are small (18–22 nucleotides) non-coding RNA molecules that can regulate gene expression at the post-transcriptional level by inhibiting mRNA translation and/or destroying the complete structure of mRNA [17,18]. miRs exert various biological functions in a cell context dependent manner, including cell proliferation, growth, and differentiation [17,18]. Up-regulation or down-regulations of certain miRs have been implicated in various disease settings [19-21], and some miRs may serve as key factors for pathogenesis. miR-21 was reported to stimulate the fibrogenic effects of hepatic stellate cells in liver, cardiac tissue and cancer-related fibrosis [22-24]. It is worth noting that miR-21 could be a key regulator of fibrosis of several organs [22,23,25-28].

First, we found that miR-21 expression was significantly upregulated after SCI. Considering the pathological process within the lesion area after SCI is mainly fibrotic, we postulated miR-21 as a key factor for regulating astrogliosis and astrocytic scar formation, as well as fibrotic scar formation. Even though miR-21 was already reported to regulate the astrocytic response [29], the mechanisms underlying its response remain unknown. Further, the *in vivo* effects of miR-21 are still yet to be defined. Also the effects of miR-21 in TGF-β signaling still need further explanation. In the current study, we investigated the potential role of miR-21 in astrocytic scar formation during the early stage of SCI *in vitro* and *in vivo*. Although both astrocytic scarring and miR-21 are controversial in this area, we identified miR-21 as a main regulator controlling fibrosis and astrocytic scar formation during the early stage of SCI. Phosphatase and tensin homologue deleted on chromosome 10 (PTEN) is a reported target protein of miR-21 and an inhibitor of the phosphoinositide 3-kinase/protein kinase B/mechanistic target of rapamycin (PI3K/Akt/mTOR) signaling pathway [27,30]. Interestingly, TGF-β1 can activate PI3K/Akt/mTOR signaling. Thus, we hypothesized that TGF-β1 may activate this pathway by regulating miR-21 expression and the interaction between miR-21 and TGF-β1 could be important after SCI. In our study, we found that miR-21 regulated astrocyte proliferation, secretion, and activation, and promoted nerve function recovery after SCI *in vitro* and *in vivo*. Although miR-21 can promote secretion of CSPGs, which inhibit axon regeneration, it can also promote the secretion of potentially beneficial factors such as BDNF and NGF. Additionally, we verified that miR-21 modulated astrocytic scarring through the PI3K/Akt/mTOR signaling pathway. Collectively, we demonstrate that miR-21 acts as a positive factor for SCI recovery in the acute phase by regulating astrocyte function through PTEN-mediate targeting of PI3K/Akt/mTOR signaling.

## RESULTS

### Altered miR and mRNA expression after SCI *in vivo.*

**Figure 1:**
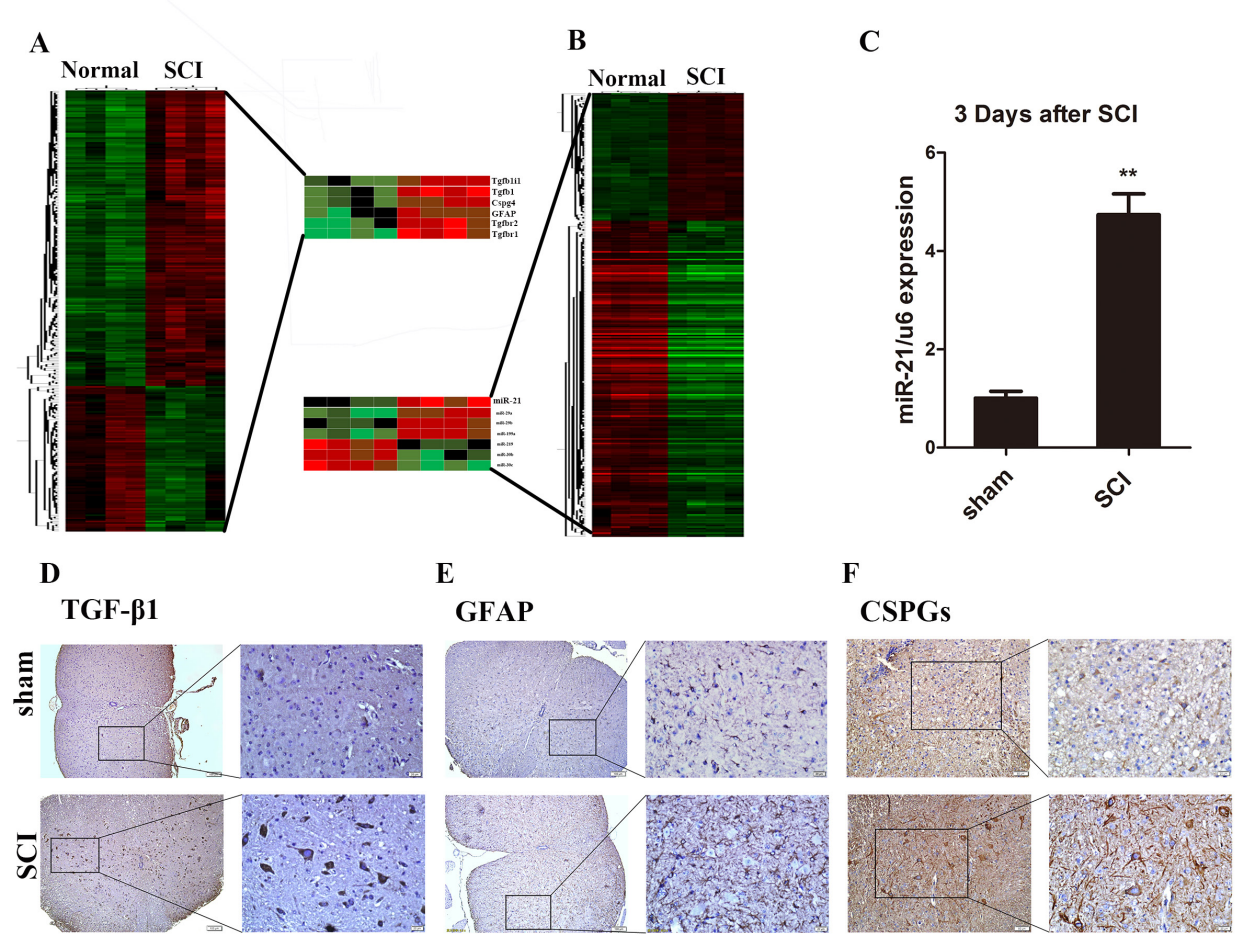
**Astrocytes were activated and miR-21, TGF-β1, and CSPGs were upregulated after SCI.** (**A**) Heat map of miRs with significantly altered expression in 3 days after SCI group compared with the sham group (n = 4). (**B**) Heat map of mRNAs with significantly altered expression in the 3 days after SCI group compared with sham group (n = 4). (**C)** qRT-PCR for miR-21 expression in sham and 3 days after SCI groups (n = 3). Data are expressed as mean ± SD. **P < 0.01 compared with sham group. Expression of TGF-β1 (scale bar: low magnification, 100μm; high magnification, 20μm) (**D**), GFAP (scale bar: low magnification, 100μm; high magnification, 20μm) (**E**) and CSPGs (scale bar: low magnification, 50μm; high magnification, 20μm) (**F**) in sham and 3 days after SCI group (n = 3). CSPGs, chondroitin sulfate proteoglycans; GFAP, glial fibrillary acidic protein; miR, microRNA; qRT-PCR, quantitative real-time polymerase chain reaction; SCI, spinal cord injury; SD, standard deviation; TGF-β1, transforming growth factor beta 1.

To gain the expression profiling of miR and mRNA after SCI, we performed the microarray analysis between tissues the sham (normal) group and day 3 post-SCI group. This screening identified 1213 upregulated mRNAs and 2008 downregulated mRNAs in SCI group as compared to the sham group. In addition, we found 93 upregulated and 189 downregulated miRs in the SCI group compared with the sham group (Fig. 1A and B). After verification and we hypothesized that miR-21 might serve as an important candidate of astrogliosis regulator, as it was significantly upregulated after SCI. The upregulation of miR-21 expression in tissues 3 days after SCI was further confirmed by qRT-PCR analysis as compared with the sham group (Fig. 1C). Meanwhile, the mRNA screening also suggested many altered gene expressions such as TGF-β1, GFAP, and CSPGs (Fig. 1B). Furthermore, the immunohistochemistry analysis confirmed the significantly elevated protein levels of TGF-β1 (Fig. 1D), GFAP (Fig. 1E), and CSPGs (Fig. 1F) in the SCI sections compared to the sham counterparts.

### miR-21 regulates astrocyte activation and function *in vitro*.

**Figure 2:**
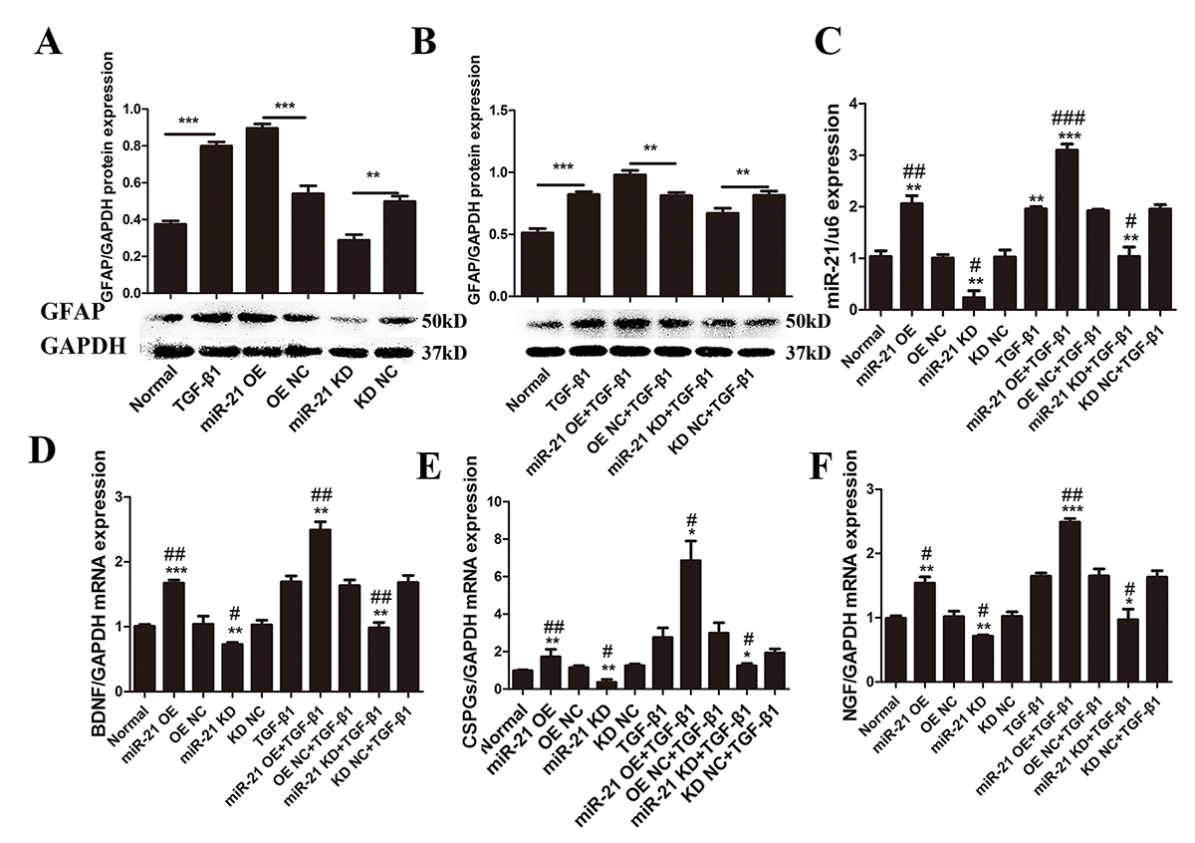
**miR-21 regulates astrocyte activation and secretion of CSPGs, NGF, and BDNF.** To determine the effects of miR-21 and TGF-β1 on astrocytes, we examined GFAP expression with or without miR-21 and TGF-β1 (10 ng/mL). (**A**) Astrocytes were treated with PBS alone or in combination with transfection of miR-21 OE, miR-21 KD, or NC (n = 3). (**B**) Astrocytes were treated with TGF-β1 alone or in combination with transfection of miR-21 OE, miR-21 KD, or NC. GFAP protein expression was examined by western blot (n = 3). (**C**) Effects of transfection were verified by qRT-PCR (n = 3). To determine changes in secretory function influenced by miR-21 and TGF-β1, astrocytes were treated with PBS alone or in combination with transfection of miR-21 OE, miR-21 KD, and NC (n = 3). RNA expression levels of BDNF (**D**), CSPGs (**E**), and NGF (**F**) were detected by qRT-PCR (n = 3). Data are expressed as mean ± SD. *P < 0.05, **P < 0.01, ***P < 0.001 compared with NC or TGF-β1 groups; #P < 0.05, ##P < 0.01, ###P < 0.001 compared with NC group. BDNF, brain-derived neurotrophic factor; CSPGs, chondroitin sulfate proteoglycans; GFAP, glial fibrillary acidic protein; miR, microRNA; NGF, nerve growth factor; miR-21 KD, LV-mmu-miR-21a-inhibition; miR-21 OE, LV-mmu-miR-21a; NC, negative control; qRT-PCR, quantitative real-time polymerase chain reaction; SCI, spinal cord injury; SD, standard deviation; TGF-β1, transforming growth factor beta 1.

As previously suggested, TGF-β1 plays a key role during astrogliosis. After treatment with TGF-β1 during the astrocytes culture, significantly upregulated GFAP expression was observed (Fig. 2A and B). In addition, astrocytes stimulated by TGF-β1 exhibited increased miR-21 expression compared with the control group (Fig. 2C). To determine whether miR-21 can regulate astrocyte activation and function, lentiviral vectors were used to manipulate miR-21 expression levels in astrocytes. After transfection, we first confirmed that miR-21 lentiviral vectors worked efficiently as compared with the negative control (NC) group (Fig. 2C). We observed that TGF-β1 can promote miR-21 expression, with the highest miR-21 expression observed in the group treated with a combination of TGF-β1 and miR-21 overexpression (OE) (Fig. 2C). Under these conditions, GFAP was also examined by western blot and immunofluorescence to evaluate the astrocyte activation and hypertrophy. Indeed, we observed the upregulated GFAP expression upon stimulation with TGF-β1 or miR-21 OE (Fig. 2A and B). In contrast, GFAP expression was downregulated upon knockdown (KD) of miR-21 expression, whether or not astrocytes were treated with TGF-β1 (Fig. 2B). Altogether, these findings strongly suggested that miR-21 could be a downstream regulator of TGF-β1 signaling and the activation of astrocytes. Additionally, qRT-PCR analysis indicated that the expression of CSPGs, BDNF, and NGF could be enhanced by miR-21 and reduced by inhibiting miR-21 expression (Fig. 2E). Thus, we speculated that increased CSPGs might result from enhanced secretion by activated astrocytes. Moreover, BDNF and NGF showed significant correlation with miR-21, indicating miR-21 might control the secretion of these nerve-related factors (Fig. 2D and F).

### miR-21 regulates astrocyte proliferation and apoptosis *in vitro*.

**Figure 3:**
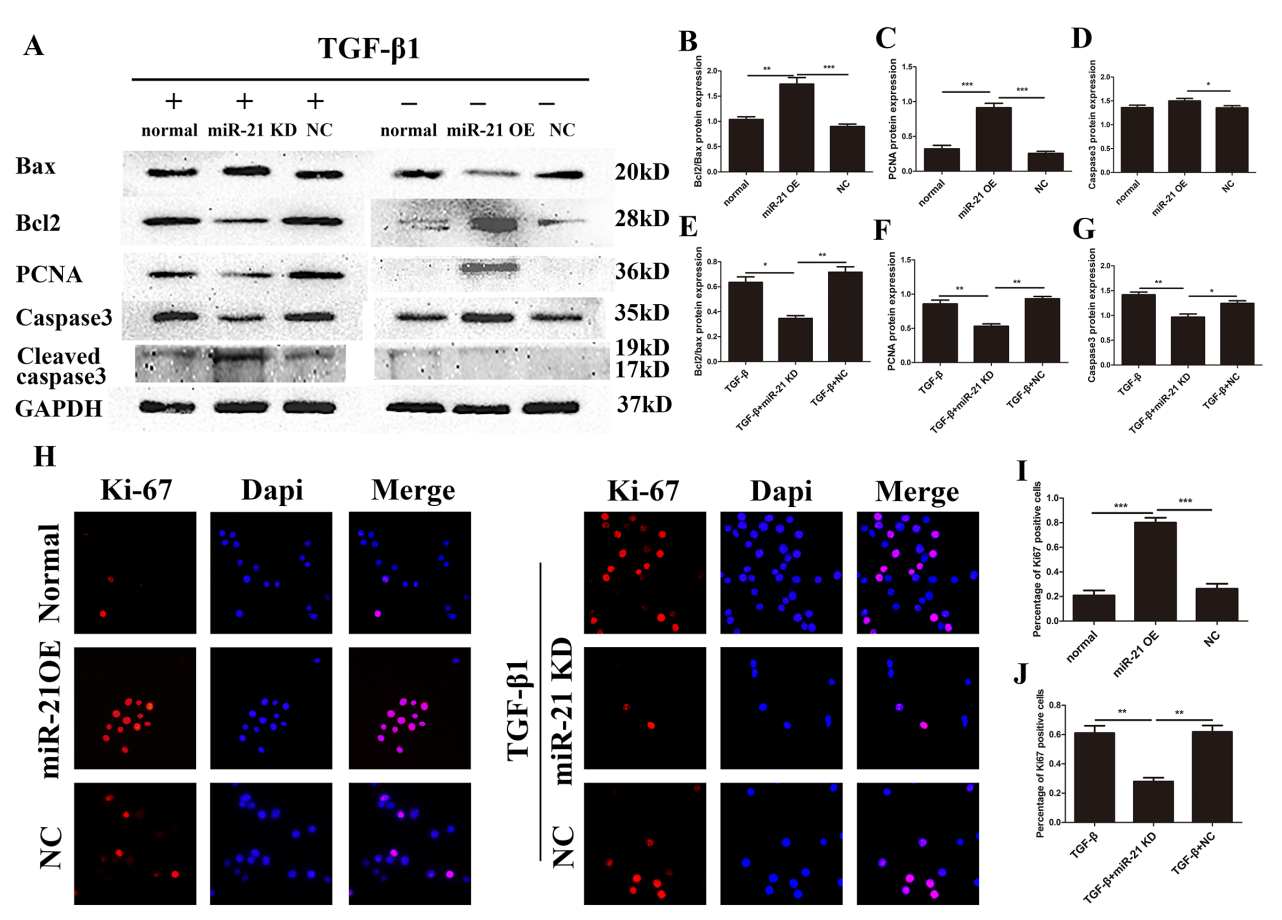
**miR-21 regulates the proliferation and apoptosis of astrocytes.** Western blot and immunofluorescence of cells divided into two groups: one treated with PBS alone, and transfected with miR-21 OE or NC; and a second treated with TGF-β1 alone in combination with transfection of miR-21 KD or NC. (**A**) Bax, Bcl2, PCNA, caspase 3, cleaved caspase 3, and GAPDH were examined by western blot (n = 5). (**B–G**) Results were analyzed by ImageJ. (**H**) Ki-67 was examined with immunofluorescence. (**I-J**) The results of immunofluorescence were analyzed by imageJ and SPSS. Data are expressed as mean ± SD. *P < 0 .05, **P < 0.01, ***P < 0.001 compared with normal, TGF-β1, or NC group. Bcl2, B-cell lymphoma 2; GAPDH, glyceraldehyde 3-phosphate dehydrogenase; miR, microRNA; miR-21 KD, LV-mmu-miR-21a-inhibition; miR-21 OE, LV-mmu-miR-21a; NC, negative control; PBS, phosphate-buffered saline; PCNA, proliferating cell nuclear antigen; SD, standard deviation; TGF-β1, transforming growth factor beta 1.

While we established that miR-21 could control astrocyte activation and secretion, whether miR-21 can regulate their proliferation and apoptosis remained unknown. Western blot and immunofluorescence were performed to examine the expression of the apoptotic proteins Bax, Bcl2, PCNA, caspase 3 and cleaved caspase 3, as well as GAPDH (Fig. 3A), which was analyzed by Image J (Fig. 3B–G). Indeed, both miR-21 and TGF-β1 promoted proliferation and inhibited the apoptosis of astrocytes. In this regard, they play a positive role after SCI, similar to previous studies examining the effect of TGF-β1 on neural regeneration [31,32]. Notably, the proliferation induced by TGF-β1 could be reduced by miR-21 KD, whereas the apoptosis can be enhanced by miR-21 KD. Similarly, the expression of proliferation related protein Ki-67, as detected by immunofluorescence (Fig. 3H), was also increased which further confirmed the results of western blots (Fig. 3I and J). Taken together, miR-21 and TGF-β1 act as important regulators of astrogliosis, and miR-21 may be a key factor for TGF-β1–induced astrogliosis.

### Upregulated miR-21 expression promoted functional recovery i*n vivo.*

**Figure 4:**
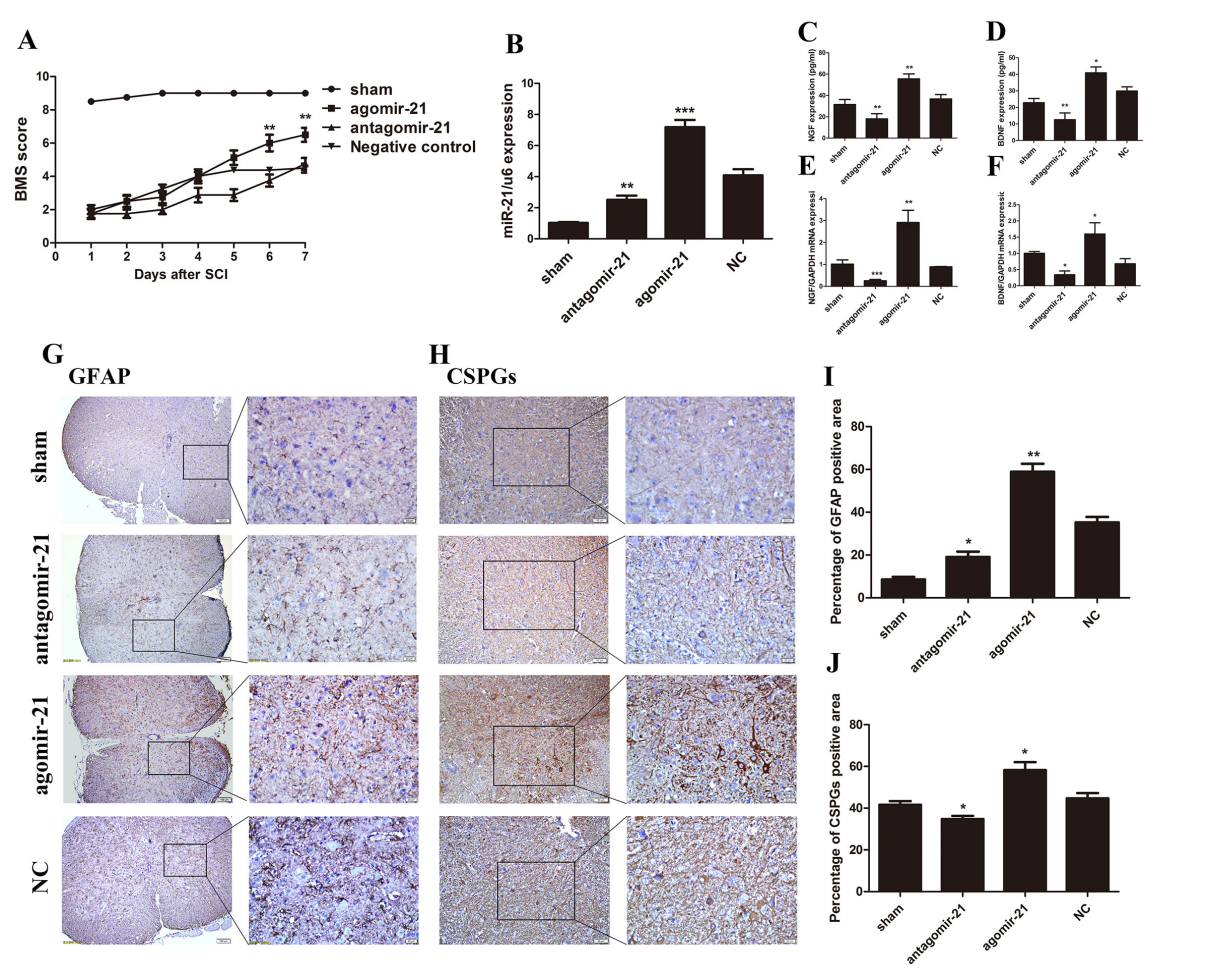
**miR-21 has a positive effect on SCI *in vivo*.** To determine whether miR-21 could regulate astrocytes *in vivo*, expression of miR-21 was interrupted in a SCI mouse model. Mice were divided into four groups: sham, agomir-21, antagomir-21 and NC. (**A**) BMS score for each group (n = 8). Unpaired t-test was used for comparison of agomir-21 and antagomir-21 groups with the antagomir NC group. Univariate analysis of variance also used to analyze this result showed that miR-21 had significant correlation with BMS scores (P < 0.001). (**B**) The expression of miR-21 was detected by qRT-PCR. BDNF (**D, F**), and NGF (**C, E**) were detected by qRT-PCR and Elisa (n = 3). Immunohistochemistry was performed to examine GFAP (scale bar: low magnification, 100μm; high magnification, 20μm) (**G**) and CSPGs (scale bar: low magnification, 50μm; high magnification, 20μm) (**H**) and the results were analyzed by ImageJ and SPSS (**I** and **J**). Data are expressed as mean ± SD. *P < 0.05, **P < 0.01, ***P < 0.001 compared with the NC group. BDNF, brain-derived neurotrophic factor; BMS, Basso Motor Score, CSPGs, chondroitin sulfate proteoglycans; GFAP, glial fibrillary acidic protein; miR, microRNA; NGF, nerve growth factor; NC, negative control; qRT-PCR, quantitative real-time polymerase chain reaction; Elisa, enzyme-linked immunosorbent assay; SCI, spinal cord injury.

To determine whether miR-21 can act positively after SCI *in vivo*, antagomir-21, agomir-21, and its negative control (NC) were used to modulate the miR-21 expression level in a mouse SCI model. We first confirmed an efficient up or down regulation of miR-21 expression by qRT-PCR (Fig. 4B). Basso mouse scale (BMS) score was also recorded every day, the scores indicated that agomir-21 promoted functional recovery compared with its NC, whereas the antagomir-21 showed the opposite effect. The agomir-21 group started to show a higher score at 5 days after SCI, which appeared to be more significant 7 days after SCI (Fig. 4A). Univariate analysis of variance showed that the functional recovery was closely related with treatment. To specify the influence of miR-21 on the secretory functions after SCI, qRT-PCR and Elisa were used to measure NGF, and BDNF. Indeed, both NGF and BDNF expression could be inhibited by treatment with antagomir-21 and induced by agomir-21; whereas ELISA analysis showed the same tendency (Fig. 4C–F). In addition, we measured the GFAP and CSPGs expression 7 days after SCI by immunohistochemistry (Fig. 4G and H), which showed that GFAP and CSPGs were downregulated after inhibiting miR-21 expression and upregulated by agomir-21. Altogether, these data indicate that miR-21 could regulate functional recovery after SCI by controlling astrocyte secretion and astrogliosis *in vivo*.

### miR-21 regulates astrogliosis through the PI3K/Akt/mTOR pathway.

**Figure 5:**
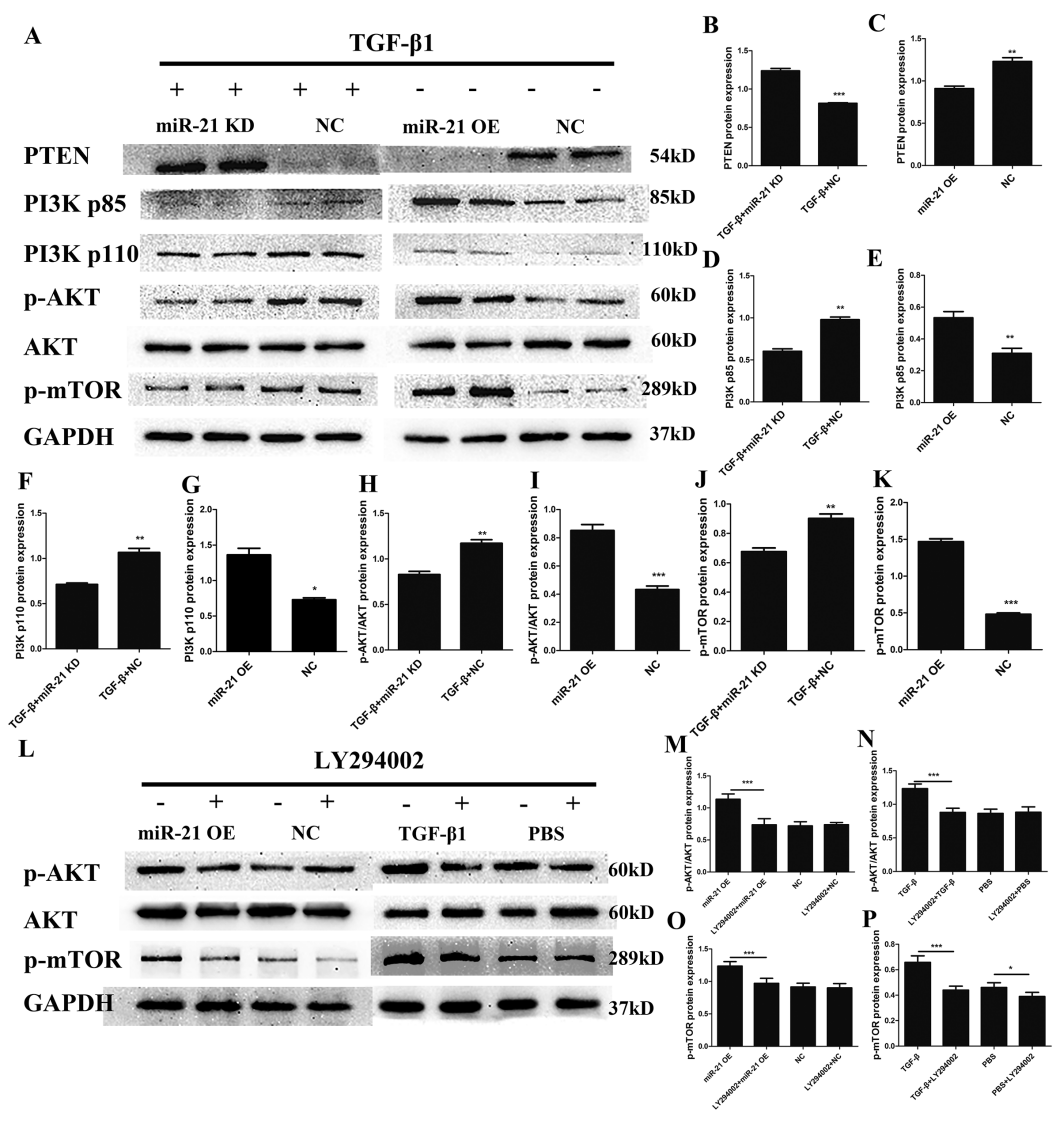
**miR-21 regulates astrogliosis through the PI3K/Akt/mTOR signaling pathway.** Western blot was performed to examine changes in the expression of key proteins in the PI3K/Akt/mTOR signaling pathway; an Akt signaling inhibitor was used to confirm results. Two groups were examined: one treated with TGF-β1 in addition to transfection with miR-21 KD or NC, and a second transfected with miR-21 OE or NC alone. (**A**) PTEN, PI3K, p-Akt, Akt, p-mTOR, GAPDH were examined (n = 5). (**B–K**) Results were analyzed by ImageJ and SPSS. Data are expressed as mean ± SD. *P < 0.05, **P < 0.01, ***P < 0.001 compared with NC group. (**L**) Two groups were analyzed: one transfected with miR-21 OE or NC with or without LY294002; and a second treated with TGF-β1 or PBS with or without LY294002. p-Akt, Akt, p-mTOR and GAPDH were examined by western blot (n=5). (**M–P**) Results were analyzed by ImageJ and SPSS. Data are expressed as mean ± SD. *P < 0.05, **P < 0.01, ***P < 0.001. Akt, protein kinase B; GAPDH, glyceraldehyde 3-phosphate dehydrogenase; miR, microRNA; miR-21 KD, LV-mmu-miR-21a-inhibition; miR-21 OE, LV-mmu-miR-21a; mTOR, mechanistic target of rapamycin; NC, negative control; PBS, phosphate-buffered saline; PI3K, phosphoinositide 3-kinase; PTEN, phosphatase and tensin homolog deleted on chromosome ten; SD, standard deviation; TGF-β1, transforming growth factor beta 1.

**Figure 6:**
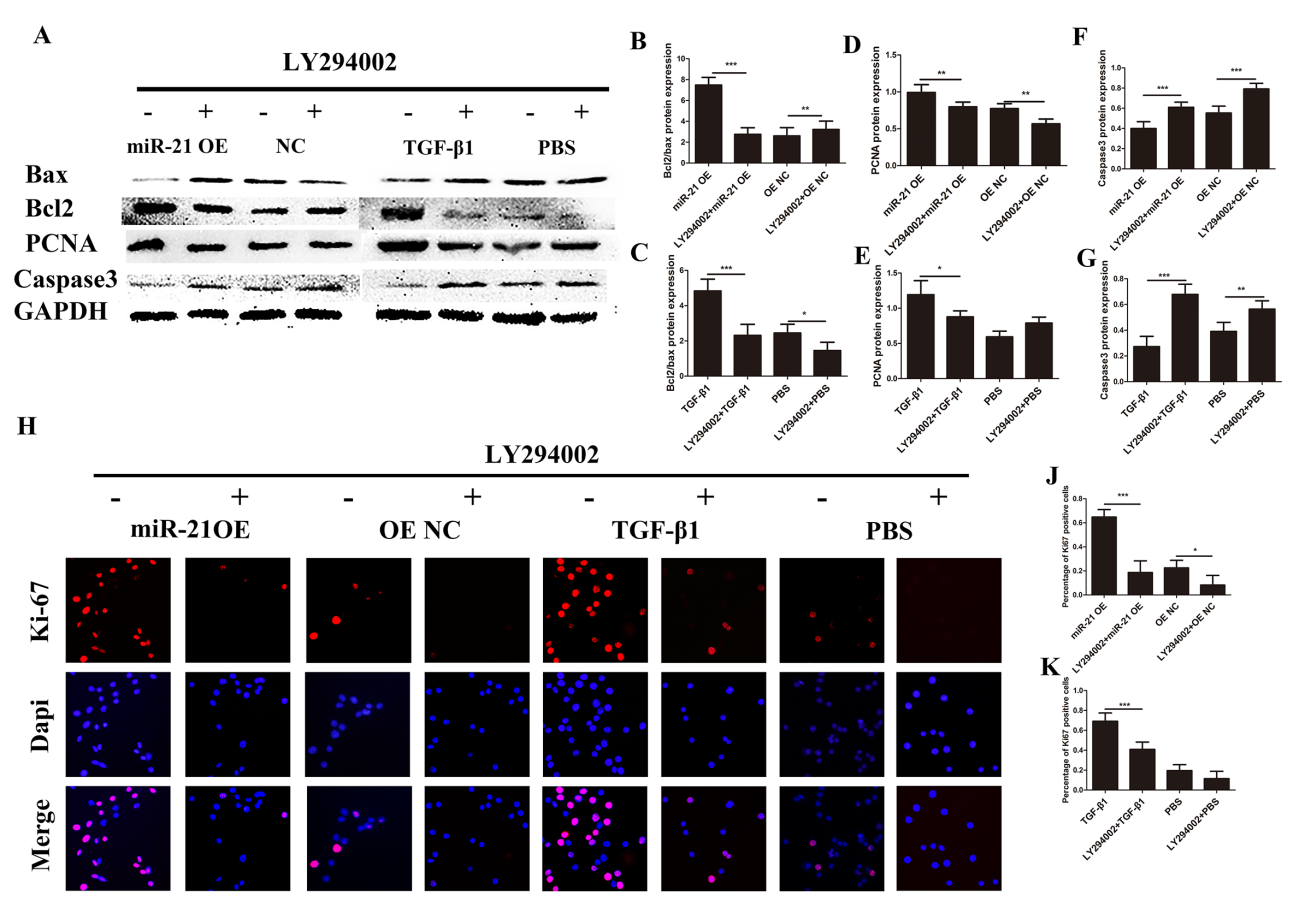
**Astrogliosis could be regulated though intervention of PI3K/Akt/mTOR signaling.** Western blot and immunofluorescence two groups: one transfected with miR-21 OE or NC with or without LY294002; and a second treated with TGF-β1 or PBS with or without LY294002. (**A)** Bax, Bcl2, PCNA, caspase 3, and GAPDH were evaluated (**B–G**) and analyzed by ImageJ and SPSS. (**H and I)** Immunofluorescence was used for the detection of Ki-67 (**H**). (**J-K)** The results were analyzed by ImageJ and SPSS. Data are expressed as mean ± SD. *P < 0.05, **P < 0.01, ***P < 0.001. Akt, protein kinase B; Bcl2, B-cell lymphoma 2; GAPDH, glyceraldehyde 3-phosphate dehydrogenase; GFAP, glial fibrillary acidic protein; mTOR, mechanistic target of rapamycin; PCNA, proliferating cell nuclear antigen; PI3K, phosphoinositide 3-kinase; SD, standard deviation.

The PI3K/Akt/mTOR pathway has been well documented to play critical roles in regulating cell proliferation, growth and apoptosis in multiple organ systems [33,34]. To explore whether the PI3K/Akt /mTOR pathway was involved in the regulatory mechanisms of miR-21 for astrogliosis, we first examined the protein expression of key proteins in the PI3K/Akt/mTOR pathway by western blot (Fig. 5A) and the expression level was quantified with Image J (Fig. 5B–K). This signaling pathway as considered as a major signaling for cell proliferation and growth. Indeed, the phosphorylation of the key components of the PI3K/Akt/mTOR pathway was upregulated with miR-21 overexpression. Collectively, these results showed that miR-21 regulated the expression/activity of the key proteins of the PI3K/Akt/mTOR pathway, which is highly involved in both cell proliferation and apoptosis. To further confirm whether this signaling pathway was affected by miR-21 and/or involved in astrogliosis, the Akt signaling inhibitor LY294002 [30] was used to decrease p-Akt expression (Fig. 5L–P). Western blot and immunofluorescence experiments demonstrated that LY294002 could reduce miR-21¬– and TGF-β1–induced proliferation and promote apoptosis, as indicated by Bax, Bcl2, PCNA, and caspase 3 expression (Fig. 6A-G). Finally, the results of immunofluorescence showed that LY294002 could also inhibit Ki-67 expression (Fig. 6H), indicating that the PI3K/Akt/mTOR pathway takes part in the regulation of miR-21.

### miR-21 regulates astrocyte proliferation and apoptosis *in vivo* through AKT/mTOR pathway.

**Figure 7:**
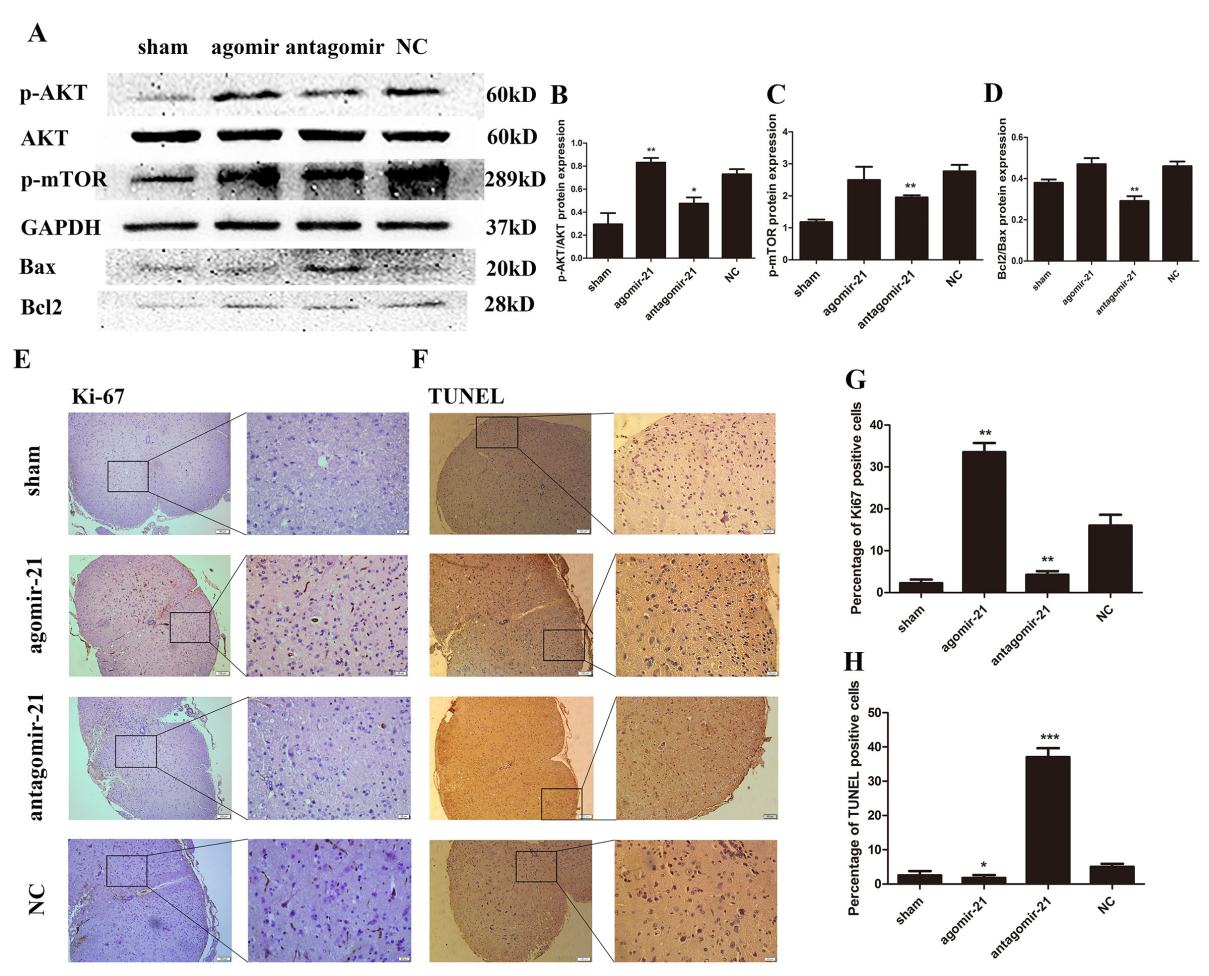
**miR-21 regulates astrogliosis through the Akt/mTOR signaling pathway.** To determine the effects of miR-21 in the regulation of proliferation and apoptosis *in vivo*, expression of miR-21 was interrupted in a SCI mouse model. Mice were divided into four groups: sham, agomir-21, antagomir-21 and NC. (**A**) p-AKT, AKT, p-mTOR, Bax and Bcl2 were detected by Western blot and analyzed by ImageJ and SPSS (**B-D**). (**E**) the expression of Ki-67 was detected by immunohistochemistry (scale bar: low magnification, 100μm; high magnification, 20μm) and analyzed by ImageJ and SPSS. (**G**). (**F**) TUNEL assay (scale bar: low magnification, 100μm; high magnification, 20μm) was performed and analyzed by ImageJ and SPSS (**H**). Data are expressed as mean ± SD. *P < 0.05, **P < 0.01, ***P < 0.001 compared with the NC group. miR, microRNA; NGF, nerve growth factor; NC, negative control; SCI, spinal cord injury.

To confirm these *in vitro* findings, Western blot was carried out to detect the protein expression of AKT, p-mTOR, Bax and bcl2 in astrocytes isolated from lesion tissue (Fig. 7A). These results were then analyzed by Image J (Fig. 7B-D). The agomir-21 can active the AKT and mTOR signaling and the antagomir-21 showed an opposite effect. Accordingly, antagomir-21 can inhibit the proliferation of the cells from tissue. On the other hand, Ki-67 was detected by immunohistochemistry (Fig. 7E) and the analysis showed an apparent difference. TUNEL assay also showed the same trend with *in vitro* studies (Fig. 7F and H). These data clearly support the notion that agomir-21 can promote the proliferation and inhibit the apoptosis of the cells in spinal cord tissue through modulating the AKT/mTOR signaling.

## DISCUSSION

Axonal regeneration and functional recovery are important for the prognosis of SCI. During this process, astrocytes have been well demonstrated to play a significant role [3]. In the present study, we demonstrated that astrocytes became proliferating, and hypertrophied after SCI, and concurrently secreted several nerve growth factors; these features were positively correlated with neural regeneration. Even though our data indicated that astrocytes could also secrete CSPGs, which are proven inhibitors of neural regeneration [2]. The recent research proved that astrocytes are not responsible for main accumulation of CSPGs [3]. So we believed that astrocytes could act as a good role in the acute phase of SCI.

**Figure 8:**
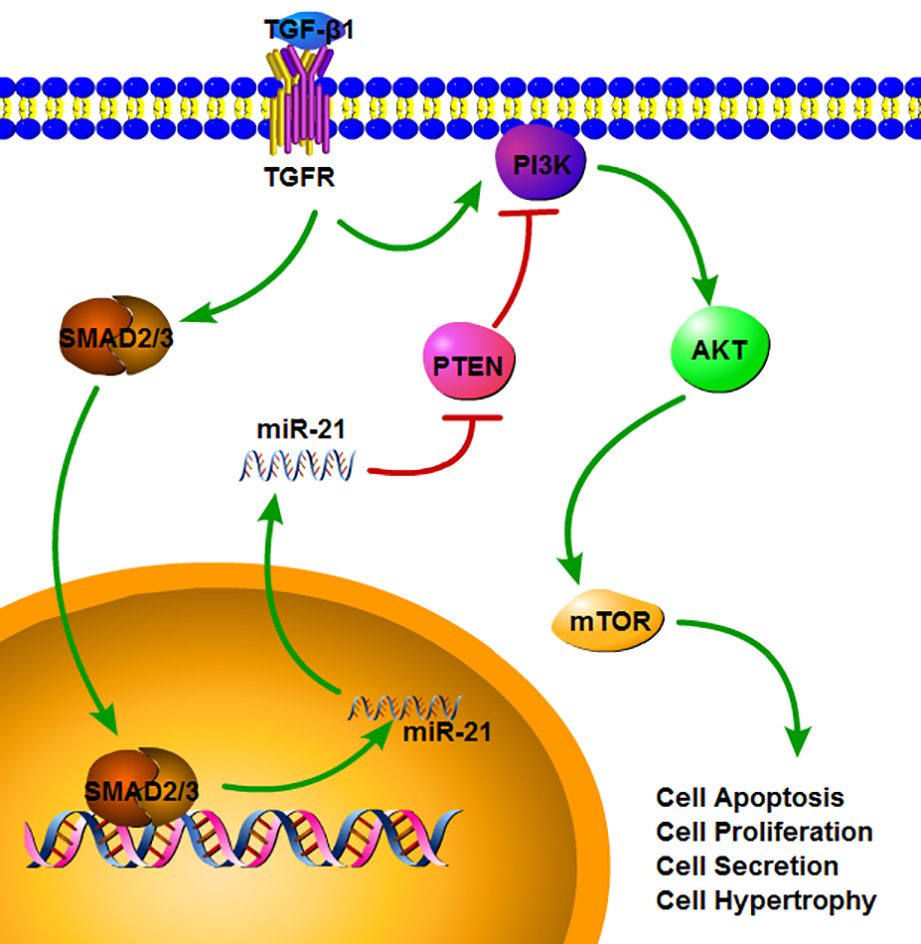
**The signaling pathway which miR-21 can regulate multiple functions in astrocytes.** TGF-β1 can up-regulate the miR-21 level through binding to TGF-β receptor and SMADs signaling. SMAD2/3 can translocate into nucleus and promote the transcription and maturation of miR-21. After the miR-21 come into the cytoplasm, it can activate the PI3K/Akt/mTOR signaling through inhibiting the PTEN expression. Also TGF-β1 can activate the PI3K/Akt/mTOR signaling, but this can be regulated by miR-21.

In our study, we found that miR-21 could act as an important regulatory factor in the acute phase of SCI. A recent study also indicated the elevated expression of miR-21 during the subacute and chronic phases [28]. TGF-β1, a key cytokine for fibrosis with increased expression after SCI, promoted astrocyte activation and miR-21 expression. We further demonstrated that the increased miR-21 expression was mediated by TGF-β1/ SMADs signaling [26], which is also highly relevant in fibrosis (as shown in Fig.8). By inhibiting miR-21 expression, the astrocyte activation induced by SCI or TGF-β1 could be suppressed. Thus, miR-21 could be a downstream factor of TGF-β1 signaling and might play a vital role in regulating the astrocyte activation, proliferation, hypertrophy and secretion capacity. The PI3K/Akt/mTOR pathway, which is implicated in cell growth, autophagy, proliferation, and other cellular functions, was involved in this process. Although a previous study found that miR-21 regulated the proliferation and apoptosis of spinal cord neurons through the PTEN/mTOR pathway [28], they did not examine the detailed underlying mechanism. By inhibiting this signaling, the proliferation and secretion induced by miR-21 and TGF-β1 could be weakened. PTEN, which can suppress this signaling, was proven to be a target gene of miR-21 [27,30]. Thus, we proposed that miR-21 could regulate astrogliosis through the PI3K/Akt/mTOR pathway by targeting PTEN (as shown in Fig.8). Since we have only examined the acute phase of SCI *in vivo*, additional studies are definitely needed to verify its potential role in promoting neural regeneration.

After the acute phase of SCI, several studies have indicated that fibrotic scarring might be an important factor inhibiting axonal regeneration [16,35]. Fibrotic scar was formed by fibroblasts, and the astrocytes may promote this formation, Interestingly, our findings suggest that disrupting astrocytic scarring could regulate fibrotic scar formation, such that astrocytic scarring could be an important point in functional recovery after SCI [4,36]. Even though in the subacute and chronic phases, fibrotic scarring becomes the main barrier for axonal regeneration, it is likely to inhibit astrocytic scar formation by regulating miR-21 expression. Thus, we conclude that the astrocytic scar formation by regulating miR-21 expression during certain phases of SCI may be vital for neural regeneration.

It is known that the pathological processes after SCI are extremely complicated. In the present study, we conclude that miR-21 can regulate astrocytic scarring through modulating the PI3K/Akt/mTOR activity by targeting PTEN. Herein, we highlighted the effect of miR-21 in promoting neural regeneration after SCI. During the acute phase, astrocytic scarring was suggested as a positive factor for neural regeneration [3]. In the subacute phase, astrocytic scarring can also be beneficial by promoting the secretion of BDNF and NGF [11], however it can promote fibrotic scar formation. Thus, promoting astrogliosis by solely increasing miR-21 expression is an effective treatment for SCI in the acute phase, but this may not be the case after that. Considering the positive effects of astrocytes in the acute phase and its negative effects in the subacute phase, and the positive role of miR-21 on astrocytes, We may speculate to develop the strategies that promoting the miR-21 expression in the acute phase and suppressing miR-21 expression in the right time, may be after the acute phase of SCI, to inhibit astrogliosis, or possibly even fibroblasts, will be the main focus of our future studies.

## MATERIALS AND METHODS

### Animals and SCI model

Male C57BL/6 background adult mice, provided by the animal center of Shandong University (Jinan, China), were maintained in individual cages under controlled conditions of 22–24°C, relative humidity of 40–60%, with a 12-h light/dark cycle and free access to food and water. The operation of this study was performed in accordance with National Institutes of Health (NIH) guidelines and regulations of the Shandong University Committee for the Care and Use of Laboratory Animals.

Mice were anesthetized with intraperitoneal 10% chloral hydrate (3 mg/kg). Laminectomy was performed at T8–T10 of the thoracic vertebra without any SCI. The moderate collision injury was caused by a modified Allen’s weight drop apparatus (8-g weight at 50 mm, 8 g × 50 mm) knocking on the exposed spinal cord. The sham group was only subjected to laminectomy without the collision injury.

### Animal experimental design and behavioral observation

Part 1. Sham and 3 days post-SCI mice groups were used for miR and mRNA microarray analysis. Twenty mice were randomly divided into two groups: sham and 3 days post-SCI (n = 4). Subsequently, microarray data were confirmed by quantitative real-time polymerase chain reaction (qRT-PCR) (n = 3) and immunohistochemistry (n = 3).

Part 2. Thirty-two mice were randomly divided into four groups: sham, antagomir, agomir, and negative control (NC) groups (n = 8 per group). In the negative control group, mice were subjected to SCI and treated intrathecally with miR-21 NC (50 μL/d, 100 nmol/mL; RiboBio, Guangzhou, China) for 3 days (0, 1, and 2 days). In antagomir and agomir groups, mice were subjected to SCI and treated intrathecally with antagomir-21 or agomir-21 (50 μL/d, 100 nmol/mL; RiboBio) for 3 days. In the sham group, mice were subjected to laminectomy without any treatment.

Locomotor activity was evaluated for Basso Motor Score (BMS) for 7 days. Two independent and well-trained investigators observed movement and scored locomotor function according to the BMS standard. Treatments were single-blind for the investigators. The final score of each animal was obtained by averaging values from both investigators. Mice were sacrificed on the seventh day by euthanasia with an overdose of 10% chloral hydrate. The injured segment was exposed and a 10-mm long spinal cord section at the injury lesion was harvested for further analysis. The mouse's blood is extracted directly from the heart and centrifuge to collect the serum for further analysis.

### miR and mRNA microarray detection

After samples were harvested and fresh-frozen in liquid nitrogen, total RNA was extracted using TRIzol™ (Invitrogen; Thermo Fisher Scientific, Waltham, MA). miR and mRNA microarray detection were performed by Shanghai Genechem (Shanghai, China).

### Cell culture and transfection

The primary cultured astrocyte from mouse (M1800-57) was purchased from ScienCell Research Laboratories (San Diego, CA). Cells were cultured in RPMI-1640 Medium (Gibco; Thermo Fisher Scientific, Shanghai, China) supplemented with 10% fetal bovine serum (Gibco; Thermo Fisher Scientific, Brisbane, Australia) and 100 IU/mL penicillin-streptomycin (Beijing Solarbio Science & Technology, Beijing, China), and were incubated in a humidified chamber supplemented with 5% CO2 at 37ºC.

LV-mmu-miR-21a (miR-21 OE) and its negative control (OE NC), as well as LV-mmu-miR-21a-inhibition (miR-21 KD) and its negative control (KD NC) were synthesized by Shanghai Genechem. The transfection was performed by adding polybrene (50 μg/mL; Shanghai Genechem) to the complete medium, which was combined with 1 x 107 TU of lentiviral vectors.

In addition, the fibrosis related factor TGF-β1 (10 ng/mL; Proteintech, Wuhan, China) was used to activate astrocytes, and the specific PI3K/Akt inhibitor LY294002 (50 μM; Selleck Chemicals, Houston, TX) was used to test the role of the Akt pathway for observed effects of miR-21.

### Protein isolation and western blot analysis

Total protein was isolated from treated astrocytes using Cell Lysate Buffer (Beyotime Institute of Biotechnology, Beijing, China) containing 1 mM phenylmethane sulfonyl fluoride and protein phosphatase inhibitor (1×; Solarbio Life Sciences, Beijing, China). Protein was collected after washing, grinding, lysis, and centrifugation. Protein concentrations were measured with a NanoDrop™ 2000/2000c Spectrometer (Thermo Fisher Scientific, Wilmington, DE). After mixing with 5× Loading Buffer (Beyotime), the mixtures were boiled in water at 100ºC for 10 min and stored at -80ºC.

Protein samples (100 μg) were separated by SDS-PAGE and transferred onto polyvinylidene difluoride membranes (Millipore, Billerica, MA). After blocking in 5% milk at room temperature for 1 h, membranes were incubated with primary antibodies at 4ºC overnight. The following day, membranes were incubated with secondary antibody for 1 h at room temperature. Images were detected by FluorChem E (ProteinSimple, San Jose, CA) and analyzed by ImageJ software (version 2.1.4.7; NIH, Bethesda, MD).

Primary and secondary antibodies were as follows: GFAP (1:1,000; Cell Signaling Technology, Danvers, MA); glyceraldehyde 3-phosphate dehydrogenase (GAPDH; 1:10,000; Wuhan Sanying Biotechnology, Wuhan, China), PTEN (1:5,000; Abcam, Cambridge, UK), PI3K P85 (1:1,000; Abcam), PI3K P110 (1:1,000; Abcam), phospho-Akt (p-Akt; 1:2,000; Cell Signaling Technology), Akt (1:1,000; Cell Signaling Technology), phospho-mTOR (p-mTOR; 1:1,000; Abcam), Bax (1:1,000; Cell Signaling Technology), B-cell lymphoma 2 (Bcl2; 1:1,000; Cell Signaling Technology), proliferating cell nuclear antigen (PCNA; 1:1,000; Cell Signaling Technology), caspase 3 (1:1,000; Cell Signaling Technology), goat anti-rabbit secondary antibody (1:5,000; Proteintech, Wuhan, China), and goat anti-rabbit secondary antibody (1:5,000; Proteintech).

### RNA extraction and quantitative real-time polymerase chain reaction

RNA was harvested from cells and tissues using TRIzol. Concentrations were determined with a Nano Drop 2000/2000c Spectrometer. Total RNA (1 μg) was used for cDNA synthesis with Takara PrimeScript RT Reagent kit (Takara Biotechnology, Dalian, China). Subsequently, expression of GFAP, CSPGs, BDNF, and NGF were detected by qPCR with a SYBR® Premix Ex Taq (Takara), using an Applied Biosystems 7300 Fast RT-PCR system (Thermo Fisher Scientific). mRNA expression levels of GFAP, CSPGs, BDNF, and NGF were normalized to GAPDH. miR-21–related qRT-PCR was performed with a Bulge-Loop™ miRNA qRT-PCR Starter Kit with miR-21 primers and U6 as an internal reference for miR-21. (RiboBio, Guangzhou, China).

Oligonucleotide primers were as follows: mouse GFAP, forward 5'-ACCAGCTTACGGCCAACAG-3' and reverse 5'-CAGCCTCAGGTTGGTTTCATC-3'; mouse CSPGs forward 5'-AGTTGGCTTCGTCAGGCACA-3' and reverse 5'-CACGCACATCACCTGGAAGTC-3'; mouse BDNF forward 5'-TCAAGTTGGAAGCCTGAATGAATG-3' and reverse 5'-CTGATGCTCAGGAACCCAGGA-3'; and mouse NGF forward 5'-TGCCAAGGACGCAGCTTTC-3' and reverse 5'-TGAAGTTTAGTCCAGTGGGCTTCAG-3'.

### Immunofluorescence

Briefly, cells in 24-well plates were rinsed twice with phosphate-buffered saline (PBS), fixed for 15 min with 4% paraformaldehyde, permeabilized for 10 min with 0.2% Triton X-100, and then blocked for 30 min with normal goat serum (Beijing Zhongshan Jinqiao Biotechnology, Beijing, China). Subsequently, cells were incubated overnight with primary antibodies at 4ºC. The following day, cells were incubated with Alexa Fluor® 594 goat anti-rabbit immunoglobulin G (IgG) secondary antibody (Thermo Fisher Scientific) for 30 min at 37ºC. After washing three times with PBS, nuclei were stained for 5 min with 4',6-diamidino-2-phenylindole (DAPI; Invitrogen; Thermo Fisher Scientific). Images were acquired with an inverted fluorescence microscope (Olympus Corporation, Tokyo, Japan). Primary antibodies were as follows: GFAP (1:1,000; Abcam) and Ki-67 (1:250; Abcam).

### Immunohistochemistry

Spinal cord sections were deparaffinized with xylene, and then hydrated using a series of 100%, 95%, and 85% ethanol solutions before heating in citrate buffer for 10 min for antigen retrieval. After washing with PBS twice, sections were incubated for 2 h at 37ºC with primary antibodies. Subsequently, sections were incubated in horseradish peroxidase-labeled goat anti-rabbit IgG secondary antibody for 30 min at 37ºC and stained with 3,3'-diaminobenzidine tetrahydrochloride (DAB). After washing with PBS for three times, sections were counterstained in hematoxylin, dehydrated, cleared, and observed with a fluorescence microscope (Olympus). Primary antibodies were as follows: GFAP (1:1,000; Abcam), Ki-67 (1:250; Abcam), and CSPGs (1:200; Abcam).

### TUNEL assay

Cleavage of genomic DNA during apoptosis may yield double stranded, low molecular weight DNA fragments (mono- and oligonucleosomes) as well as single strand breaks (“nicks”) in high molecular weight DNA, which can be measured using In Situ Cell Death Detection kit (Roche) according to the manufacturer's instructions. Briefly, the paraffin-embedded tissue sections were permeabilized after dewaxation, rehydration, protease treatment with 0.1% Triton1) X-100 in 0.1% sodium citrate. Then the samples were added in TUNEL reaction mixture and Converter-POD, Substrate solution in order. The apoptosis was then analyzed using light microscopy.

### Statistical analysis

All analyses were performed using GraphPad Prism v5.01 software (GraphPad Software, La Jolla, CA). Student's t test was used to analyze differences between two groups. One-way analysis of variance with Bonferroni’s tests was used to analyze more than two groups. All data are presented as mean ± standard deviation (SD), with mean values calculated according to at least three independent experiments. Statistically significant differences were defined at P < 0.05.

### Abbreviations
